# Research protocol: effect of natural S-equol on blood pressure and vascular function- a six-month randomized controlled trial among equol non-producers of postmenopausal women with prehypertension or untreated stage 1 hypertension

**DOI:** 10.1186/s12906-016-1065-5

**Published:** 2016-03-01

**Authors:** Zhao-min Liu, Suzanne C. Ho, Yu-ming Chen, Yao Jie Xie, Zhi-guan Huang, Wen-hua Ling

**Affiliations:** Division of Family Medicine and Primary Care, The Jockey Club School of Public Health and Primary Care, the Chinese University of Hong Kong, Hong Kong, SAR; Division of Epidemiology, The Jockey Club School of Public Health and Primary Care, the Chinese University of Hong Kong, Hong Kong, SAR; Department of Medical Statistics and Epidemiology, School of Public Health, Sun Yat-sen University, Guangzhou, PR China; School of Nursing, Faculty of Health and Social Science, the Hong Kong Polytechnic University, Hong Kong, SAR; Department of Sport and Health, Guangzhou Sport University, Guangzhou, PR China; Department of Nutrition, School of Public Health, Sun Yat-sen University, Guangzhou, PR China

**Keywords:** Equol, Supplementation, Blood pressure, Vascular function, Postmenopausal women

## Abstract

**Background:**

Although higher habitual soy intake is associated with lower blood pressure (BP) and stroke incidence, clinical trials using soy protein or isoflavones on cardiovascular risks yielded inconsistent results. The discrepancies are hypothesized to be due to the individuals’ intestinal bacterial capacity to metabolite isoflavones daidzein into equol. Animal and in vitro studies have revealed that equol has stronger estrogen-like and anti-oxidative activity than isoflavones and possesses natriuretic and vasorelaxant properties which may play an important role in the prevention of hypertension. However, no clinical trial has examined the effect of equol on BP. We thus propose a 24-week randomized controlled trial to test the effectiveness of natural S-equol on BP and vascular function among equol non-producers.

**Methods/design:**

This will be a 6-month double-blind, randomized, placebo-controlled trial among 207 non-equol producing postmenopausal women with prehypertension or early untreated hypertension. Eligible participants who have completed a 2-week run-in will be randomized to either one of the 3 groups: placebo group, low-equol group (10 mg/d) and high equol group (20 mg/d). The outcome measures will be conducted at baseline and at the end of the trial including 24 h ambulatory BP, endothelial function (by ultrasound determined brachial flow mediated dilation), arterial stiffness (by pulse wave analysis) and other cardiovascular risk factors (lipid profile, glycemic control and inflammatory biomarkers). Urinary isoflavones will be tested for compliance assessment. One way analysis of variance will be applied to compare the 6-month changes in ambulatory BP or parameters of vascular function among the 3 treatment groups.

**Discussion:**

This study will be performed in community subjects. If the antihypertensive effect of equol is proven, the provision of natural equol to those high risk adults who are unable to produce equol will have enormous public health implications for the primary and secondary prevention of hypertension and cardiovascular diseases on a population basis. The research efforts will also have significant implications for industry in the provision of suitable soy products for the prevention of hypertension and its related complications.

**Trial registration:**

The trial was registered in ClinicalTrials.gov with identifier of NCT02515682.

## Background

### Hypertension is an important public-health challenge

Hypertension is an independent risk factor for cardiovascular diseases (CVD) and renal impairment [1]. An estimated 1 billion individuals are hypertensive worldwide which is estimated to increase to 1.56 billion by 2025 [2]. The risk of CVD will double with each increment of 20/10 mm Hg (SBP/DBP) from 115/75 mm Hg onwards [3]. A meta-analysis by He et al. [4] reported that 49.5 % of strokes in a Chinese population could have been prevented if hypertension was eliminated. A reduction of systolic BP by only 2 mmHg may result in a 6 % reduction in fatal stroke and a 4 % reduction in coronary heart disease (CHD) [5]. Thus, the prevention among the early stage of hypertension will have enormous public health implications.

Prehypertension (SBP 120–139 mm Hg or DBP 80–89 mm/Hg) was the strongest predictor of incident hypertension and associated with elevated risk of incident CVD [3, 6]. Vason et al. [7] observed a 30 % conversion of prehypertension to hypertension over 4 years in the Framingham cohort study. The conversion is even higher in women than men and incident hypertension is highest in women aged above 55 y [8]. Hong Kong 2003/2004 Population Health Survey [9] reported that prehypertension has the highest prevalence of 42 % in midlife women aged 50–59y. Pre-hypertension and its progression to hypertension and increased risk of CVDs have enormous public health implications but the treatment of prehypertension is still debatable [10]. Life-style modification for prehypertension is recommended [3, 11] but compliance is generally poor. Treatment by antihypertensive drugs often produces more or less drug related adverse events, thus the use of natural remedies has aroused increasing interests.

### Vascular function assessment by endothelial function and arterial stiffness

Hypertension is associated with exaggerated functional vascular damage such as endothelial dysfunction and increased arterial stiffness [12]. Endothelial dysfunction is characterized by a reduced capacity of endothelial cells to suppress processes of inflammation, thrombosis and oxidative stress. Impaired arterial elasticity causes increased cardiac after-load, coronary arterial blood supply, atherogenesis and microvascular damage [13]. Vascular dysfunction is considered as a central pathophysiologic process in the initiation and progression of hypertension and atherosclerosis. Evidence shows lifestyle modification can help restore endothelial function (EF) and arterial elasticity. Vascular function can be detected early by several non-invasive methods such as high-resolution ultrasound and tonometry system. Brachial flow mediated dilation (FMD) and pulse wave analysis (PWA) are valuable surrogate markers of vascular function in hypertensive and other cardiovascular conditions [14].

### Soy, equol and hypertension or cardiovascular health

Soy is a traditional Asian diet. Several large scale cohort studies [15, 16] have revealed that the higher habitual soy foods or isoflavones intake is associated with lower BP and cardiovascular incidence. However, the effective components of soy and the underlying mechanisms are still unclear. Soy protein and isoflavones are the most studied components in soy with regard to potential health benefits. However, clinical trials using soy protein and/or isoflavones have yielded mixed findings on cardiovascular risks [17, 18]. The discrepancies are hypothesized to be associated with the individuals’ intestinal capacity to metabolite isoflavones daidzein (one principle isoflavone) into equol [18]. Setchell et al.[19] proposed that equol could be the key molecule accounting for the health benefits of soy or isoflavones. Equol is a metabolite of isoflaovone daizein by intestinal bacteria. Though the majority of animals produce equol, only 20-50 % human adults produce equol following soy/daidzein challenge.

Observational studies have suggested that equol production was associated with decreased risk of certain diseases or conditions such as breast cancer [20], obesity [21], menopausal symptoms [22], hypertension and vascular dysfunction [23, 24]. Our recent published data [25] among 648 prehypertensive postmenopausal women also indicated that equol producers had lower systolic BP (134.8 ± 19.3 vs 138.6 ± 24.8 mmHg, *p* = 0.028), serum triglyceride (1.29 ± 0.63 vs 1.40 ± 0.78 mmol/l, *p* = 0.023), total cholesterol (5.61 ± 0.94 vs 5.77 ± 0.90 mmol/l, *p* = 0.046) and free fatty acid (550.3 ± 200.5 vs 616.6 ± 206.1 μmol/l, *p* = 0.005) than equol non-producers.

Clinical trials have reported that the beneficial effect of soy on cardiovascular health was only present or more pronounced in equol producers than non-producers on the improvement of lipids profiles [26–28], blood pressure [28, 29], vascular function [29, 30] and inflammatory markers [30], although some did not [31, 32]. However, most of these findings are from non-prespecified subgroup analysis of equol-producers, not randomized accordingly.

Equol is currently attracting considerable interest as a potential pharmaceutical or nutraceutical agent [33]. Equol occurs as two enantiomeric forms, S-equol and R-equol, and presents in vivo only as the S-equol [19]. S-equol has a much higher apparent bioavailability (near half of it circulates in the free form) [33] and slower clearance rate (the terminal elimination half-life of 7–8 h) than its precursor daidzein [33]. Equol is superior to all other isoflavones in its estrogenic property and antioxidant activity [34, 35]. In vitro cell culture and animal studies [36–39] have demonstrated that equol possesses natriuretic and vasorelaxant properties via increasing the transcription of endothelial nitric oxide synthase (eNOS) and redox-sensitive genes, enhancing renal blood flow and sodium excretion, which plays an important role in the attenuation of the development of hypertension. To date, several clinical trials have reported favorable effects of S-equol on reducing bone resorption [40], alleviating menopausal symptoms [41, 42], improving glycemic control and reducing LDL-c [43]. However, no randomized controlled trial (RCT) has tested the effect of natural S-equol on BP and vascular function among prehypertensive population.

### Knowledge gap on equol and hypertension or vascular function

Although dozens of studies have examined the effect of soy on BP and cardiovascular health, clinical trials directly using natural S-equol as supplement are limited. Most of these studies have relatively short duration and the criteria for determining equol producing status are not consistent across all the studies. To date, no RCT has tested the effect of S-equol on 24 h ambulatory BP and vascular function among pre-hypertensive subjects. Among previous studies on the soy and cardiovascular health, BP was always taken in one or two clinic occasions with few data on ambulatory BP measurement. It has been well established that ambulatory BP (ABP) monitoring offers substantial advantages over conventional clinic measures due to a good reproducibility and precision, reducing placebo effect and excluding white-coat influence.

### The study hypotheses and purpose

To address the above limitations, we propose to perform a 24-week randomized controlled trial (RCT) among non-equol producing post-menopausal women with pre-hypertension or initial untreated hypertension to verify if natural S-equol has anti-hypertensive effects or can improve vascular function and other cardiovascular risks (lipids, glucose, and inflammatory biomarkers). To control for the influence of equol phenotype, we will limit the participants to non-equol producers since self-produced equol from habitual soy intake in equol producers may neutralize the health effect of equol supplementation. Considering a high prevalence of prehypertension in midlife Hong Kong women [9] and a relatively good compliance of intervention in this age group in our previous trial [44], the study will be conducted in postmenopausal women. We hypothesized that natural S-equol could decrease BP and improve vascular function in equol non-producers of postmenopausal women with untreated prehypertension or early hypertension. There is a dose–response effect with higher dosage of natural S-equol producing more prominent effects on these health outcomes than that of low dose.

## Method/design

This study will be a 6-month double-blind, randomized, placebo-controlled trial with three intervention arms.

### Participants

Our recently completed RCT [45] has screened out 292 equol non-producers of postmenopausal women with untreated prehypertension or hypertension. Subjects have been recruited from community or health assessment center via advertisements in newspaper or referrals. They were initially screened using a standardized questionnaire and their eligibility will be further confirmed at research centre based on the pre-defined criteria.

### Inclusion criteria

Hong Kong Chinese women aged 45–65 y with 2 ~ 8 years menopausal; mean SBP above 130 mmHg or DBP above 80 mmHg or both based on an average of 6 BP readings on two different occasions measured by sphygmomanometer. Equol non-producer is defined as 24-hour urinary log_10_*S-equol*:daidzein ratio less than −1.75 after daidzein challenge (60 mg daidzein daily for 7 consecutive days) [46]. Written informed consent will be obtained from all the participants prior to enrolment. Ethics approval has been obtained from the Clinical Research Ethics Committee of the Chinese University of Hong Kong (CREC Ref. No.: CRE-2013.119-T).

### Exclusion criteria

Subjects on anti-hypertensive medication or with average SBP ≥ 160 or DBP ≥ 100 or both; use of medications known to affect BP within past 6 months (hormone therapy, hypoglycemic or weight reduction agents); medical history or presence of certain chronic diseases (stroke, cardiac infarction, severe liver and renal disease etc.) that could affect BP or limit the individual’s ability to participate in the study; present or history of breast cancer, endometrial cancer, ovarian cancer, thyroid disorder, abnormal uterine bleeding after menopause; on diet for weight loss, glucose or cholesterol reduction or vegetarian diet; regular smoker or alcohol consumption more than 30 g/day; known soy allergy.

### Sample size planning

Our previous RCT [47] on soy showed a 4.2 mmHg net reduction (SD of change 10 mmHg) in SBP after 6-month soy protein and isoflavones supplementation relative to placebo group among 130 pre- and hypertensive postmenopausal women [48]. Assuming a reduction of 5 mmHg of treatment effect on SBP (main effect), 63 subjects per group will yield a power of at least 80 % at 5 % level of significance (for a 2-side t-test). Allowing for 10 % non-compliance or drop out from the study, 69 subjects per group and a total 207 subjects (63 × 3 × 110 %) will be recruited. Similar attrition rates (7.8 % and 7.0 %) have been reported in our previous soy trials [45, 49].

### Intervention regimes and supplement preparation

Two hundred seven eligible equol-non-producers of post-menopausal women who have completed a 2-week run-in period will be randomized to either one of the 3 groups; placebo group, low-equol group (10 mg/d) and high equol group (20 mg/d).

**Table:** 

Groups	Supplementations			Sample size	Duration
	S-equol	Starch	soy flour		
Placebo	0	20 mg	10 g	69	6-month
Low-equol	10 mg	10 mg	10 g	69	6-month
High-equol	20 mg	0	10 g	69	6-month

The dose of natural S-equol used in the present study is determined mainly on previous studies. A 10 mg natural S-equol is reported to be effective for improving menopausal symptoms [41] and bone turnover [40] with no observed side effect. A higher dose of 30 mg/d for 12-week [42] or even 160 mg/d for 14-day [50] in previous pilot studies did not reveal any serious adverse events except for one woman developing a rash [42]. The safety of S-equol has been confirmed in genotoxicity [51], acute and subchronic toxicity [52], and reproductive and development toxicity tests [53].

In order to obtain good blinding effect and improve supplement solubility in water, 10 g soy flour will be included in all 3 groups. Our previous trials have shown soy product was more acceptable to mid-life women than milk powder. Starch will be added to supplements to make 3 groups of packets with similar weight. The natural S-equol will be purchased from Otsuka Pharmaceutical Co. Ltd (Japan). The supplements of 3 groups will be processed by a local GMP certified pharmaceutical company. Daily dosage will be filled into identical looking packets. The supplements will be delivered to subjects every four weeks.

Supplement will be suggested to be mixed with 100 ml of water, juice, milk or adding to soft foods such as oatmeal and intake daily before breakfast. Subjects will be asked to maintain their usual level of physical activity, discontinue the use of other dietary and herbal supplements (i.e., dietary fiber, minerals, Vitamin C and E, fish oils, flavonoids etc.) known to influence BP, minimize the intake of soy foods (≤2 servings per week), refrain from high salt diet and restrict alcohol intake (≤2 drinks per week).

### Run-in exercise

Prior to the study proper, a 2-week run-in exercise with the use of placebo supplement (20 mg starch + 10 mg soy flour), will be carried out to familiarize the subjects with the trial requirements. Subjects who consume ≥90 % of supplements provided and without any side-effects will be formally enrolled into the study.

### Randomization procedures

A block randomization will be used for subject allocation. 207 continuous serial numbers (1–207) will be divided into 23 blocks with a block size 9. In each block, three treatments will be randomly allocated for each serial number based on the sequence of computer generated random numbers by SPSS (Release 16, SPSS Inc., Chicago, USA). The 207 serial numbers will be labeled on the corresponding supplements. Each serial number will correspond to one of three treatments. The supplements will be assigned to participants according to the sequence of their visits after the completion of the run-in period. Three treatments will then be randomly allocated to the three groups.

### Blinding and code breaking procedures

The three identical looking supplements will be labeled with a serial number by a co-investigator who is not responsible for subject contact and assessment. Participants, investigators and laboratory technicians will be blinded to the treatment assignment until the conclusion of the trial. The principal investigator will be responsible for breaking the randomization code after the completion of data analyses or in emergency situations. Intensive effort will be undertaken to locate a subject who fail to attend for follow-up interview or examination. Reason for non-completion or termination will be documented.

## Data collection

Twenty-four hours ABP, vascular function, overnight fasting (10–12 h) venous blood samples and 24-hour urine samples will be measured or collected at baseline and at 24 weeks after treatment. Plasma/serum will be isolated within 2 h after collection. Specimens will be stored at −80 °C till analyses. All samples from each subject will be run in the same assay/batch to avoid inter-assay variability. Structured questionnaire interview and anthropometric measurements will be administered at baseline and at 24 weeks. Habitual dietary intakes will be evaluated by a validated food frequency questionnaire and habitual physical activity by Modified Baecke questionnaire validated in Hong Kong population. Urinary analysis for sodium, potassium, and creatinine will be tested by Hitachi 917 autoanalyzer, Roche Diagnostics.

### Primary outcome measures—24-hour ambulatory BP

All arterial parameters will be measured at baseline and 24 weeks by an experienced research assistant. Participants will be asked to fast for 8 h and withhold vasoactive medications, alcohol, and caffeine for 12 h before the study visit.

### 24-hour ambulatory BP

Twenty-four hours ABP will be measured with ABP Monitor (TM2430, A&D Company, Limited, Japan) based on a standard protocol [54]. Participants will be fitted with an ABP monitor by a trained investigator, with the cuff attached to the non-dominant upper arm for the entire 24-hour period. The measurements will be carried out every 30 min during the daytime (from 06:00 a.m. to 12:00 p.m.) and 60 min during the night (1:00 a.m. to 06:00 a.m.), providing at least 40 valid readings of BP throughout the 24-hour. Participants will be received verbal and written instructions on the monitors and complete a diary to record sleep, medication, posture activity and symptoms. A series of calibration readings will be taken with a mercury sphygmomanometer (within 5 mm Hg of the mercury readings). The mean values of SBP and DBP in all day will be calculated by the device-specific software.

### Secondary outcome measures- vascular function

#### Brachial endothelial function by flow mediated dilation (FMD)

Vascular endothelial function will be assessed with high-resolution ultrasound (SIMENS, ACUSON S2000) on the brachial artery during reactive hyperemia by standard methodology [55]. Ischemia is induced with a suprasystolic pressure cuff around the upper portion of the arm. After exactly 5 min of occlusion, reactive hyperemia will ensue and the peak brachial artery diameter will be measured between 45 and 75 s. Percentage FMD is defined as the difference between the maximal brachial artery diameter during reactive hyperemia and the baseline divided by the baseline brachial artery diameter.

#### Arterial stiffness and stenosis by pulse wave analysis (PWA)

PWA will be performed by a dedicated tonometry system (VP-2000; Omron Healthcare, Inc. Bannockburn, Illinois, USA), following a standardized procedure [56]. Patients will be fitted with sphygmomanometer cuffs on both arms and legs and a carotid artery pressure sensor on the neck. Pulse wave velocity (PWV) will be determined from recorded pressure wave forms over both the brachial-ankle and the heart-ankle arterial segments, which are derived from computer generated pulse transit times and measured distances between the two recording sites. Ankle brachial index (ABI) is the ratio of SBP of ankle to brachial reflecting lower-extremity arterial stenosis.

### Tertiary outcomes

#### Biomarkers for endothelial function and inflammation

Serum endothelin-1 (ET-1), soluble vascular cell adhesion molecule–1 (VCAM-1), soluble intercellular adhesion molecule–1 (ICAM-1) and E-selectin will be measured by commercial ELISA kits. Serum C-reactive protein (CRP) will be measured with a validated high sensitivity immunoturbidimetric assay.

### Other cardiovascular risk markers

Anthropometric measurements, plasma lipids profile and fasting glucose will be conducted at baseline and 6-month. Height, weight, waist and hip circumference, body fat percentage will be measured by standardized procedure. Body mass index (BMI), and waist to hip ratio (WHR) will be calculated. Serum/plasma glucose, triglyceride, total cholesterol, HDL and LDL cholesterol will be analyzed (Hitachi 917 auto-analyzer, Roche Diagnostics).

### Assessment of compliance

Compliance will be assessed by counting the returned supplements and estimation of percentage of intake, as well as urinary excretion of isoflavones at baseline and 24 weeks. Urinary isoflavones including daidzein and equol will be determined by high performance liquid chromatography (HPLC).

### Statistical analysis

Relevant parametric and non-parametric tests will be used for testing the differences in the baseline characteristics and outcomes of post treatment among the three study groups. The major approach of analysis will be one way analysis of variance (ANOVA) to compare the change and percentage change of outcomes at 24 weeks among the three groups. Analysis of covariance will also be made to examine if the results are similar after adjusting for potential confounders. The primary analysis will be an intention to treat (ITT) analysis that included all subjects who are randomized. A secondary per protocol analysis will be performed including subjects with good compliance (defined as subjects who consumed ≥ 80 % of provided supplements or with urinary isoflavones level above predefined values for valid users). The non-compliant subjects will be described and compared to the compliant subjects. Dose-dependent effects will be evaluated with the Jonckheere’s trend test. The Bonferroni test will be used for post hoc multiple comparisons among the three groups. The primary outcome variables of 24 h ambulatory BP (mean SBP and mean DBP in all day) will be tested at a significance level of 2.5 % in order to maintain an overall 5 % level. The two important secondary outcomes for assessment of vascular function (%FMD and PWV) will be tested at a significant level of 1.25 %. Exploratory analyses will be used to test each of the tertiary outcomes at the 5 % significance level. Further stratification analysis will be conducted to examine if the effects differed across subgroups such as years since menopause and BMI etc.

## Results

**Fig. 1:**
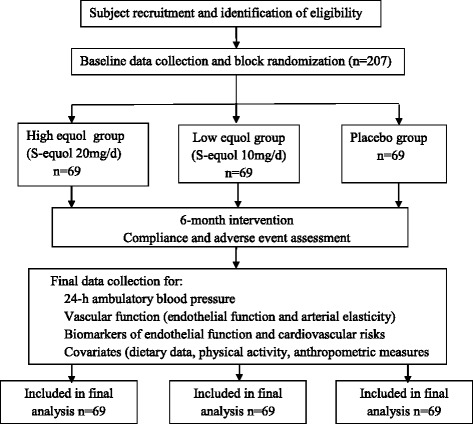
Study flow chart of the proposal

The proposed study flow chart is indicated in Fig. 1. Baseline characteristics of 292 pre-hypertensive postmenopausal women who were non-equol producers were listed in Table 1.

**Table 1:** Baseline characteristics among 292 non-equol producing postmenopausal women with prehypertension

	Non-Equol producers
n	292
Age (y)	57.6 ± 4.6
Menopausal years (y)	8.2 ± 5.3
Age at menarche(y)	13.2 ± 1.8
Ever use of contraceptives (%)	126 (50.6)
Ever use of HRT (%)	38 (15.3)
Dietary intake (from FFQ)	
Energy (kcal/d)	2100.7 ± 1018.6
Protein (g/1000 kcal)	55.8 ± 13.2
Total fat (g/1000 kcal)	21.2 ± 6.9
Fiber (g/1000 kcal)	21.0 ± 8.8
Cholesterol (mg/1000 kcal)	146.2 ± 90.1
Isoflavones (mg/1000 kcal)	7.03 ± 7.83
Total PA (MET-min/d)	1445.6 ± 728.2
Occupational PA	586.1 ± 759.5
Housework PA	558.7 ± 459.9
Sports PA	141.0 ± 166.2
Habitual tea drinking (%)	195 (78.6)
Habitual alcohol drinking (%)	18 (7.3)
Habitual coffee drinking (%)	84 (33.9)
Passive smoking (%)	43 (17.4)
Anthropometrics measures, BP and lipids	
Body weight (kg)	55.7 ± 9.0
BMI (kg/m^2^)	23.0 ± 3.2
WHR	0.838 ± 0.050
SBP (mmHg)	135.1 ± 16.6
DBP (mmHg)	83.1 ± 9.6
TG (mmol/L)	1.41 ± 0.79
TC (mmol/L)	5.76 ± 0.90
Hs-CRP (mg/L)	1.90 ± 2.08

Data are presented as mean ± standard deviation for continuous variables or number (%) for categorical variables. FFQ indicates food frequency questionnaires; PA indicates physical activity; Habitual drinking means drinking alcohol, tea or coffee more than 1 time per week; METs are multiples of resting metabolic rates and a MET-minute is computed by multiplying the MET score of an activity by the minutes performed. Dietary nutrients intakes were calculated mainly based on the China Food Composition Table 2002 and 2004. *SBP* systolic blood pressure, *DBP* diastolic blood pressure, *TG* triglycerides, *BMI* body mass index, *WHR* waist to hip ratio, *Hs-CRP* high sensitivity c-reactive protein

## Discussion

The results of this RCT will produce direct evidence on the effectiveness of natural S-equol on BP and vascular function among equol non-producing Chinese postmenopausal women with pre-hypertension or early untreated hypertension. To our knowledge, this will be the first RCT specifically conducted among non-equol producers to test the effectiveness of S-equol supplementation on BP and vascular function, and also provide evidence for the optimal dosage of equol. S-equol is natural supplement derived from soy germ by fermentation. The proposed level of intake has been used in several clinical trials. No severe adverse effects have been observed in human studies.

This randomized, double-blind, placebo-controlled trial will be performed in community subjects with prehypertension or early untreated hypertension. If the antihypertensive effect of natural S-equol is proven, the provision of natural equol to those high risk adults who are unable to produce equol due to a lack of intestinal equol-producing bacteria or some other factors, could have enormous public health implications for the primary and secondary prevention of hypertension and cardiovascular diseases on a population basis. These research efforts may provide alternative remedy to improve cardiovascular health, and also have significant implications for industry in the provision of suitable soy products or supplementation for the prevention of hypertension and its related complications. With the high prevalence of hypertension in postmenopausal women, this study on the hypotensive effect of equol will explore an area with important public health implications both locally and internationally.

### Ethics approval

The study ethics was approved by the Ethics committee of the Chinese University of Hong Kong (CREC Ref. No.: CRE-2013.119-T)

## Abbreviations

ABPM: ambulatory BP monitor
ANOVA: one way analysis of variance
BMI: body mass index
BP: blood pressure
CHD: coronary heart disease
CRP: C-reactive protein
CVD: cardiovascular diseases
EF: endothelial function
ET-1: endothelin-1
FMD: flow mediated dilation
HPLC: high performance liquid chromatography
ICAM-1: soluble intercellular adhesion molecule–1
PWA: pulse wave analysis
RCT: randomized controlled trial
VCAM-1: soluble vascular cell adhesion molecule–1
WHR: waist to hip ratio
